# Association of the inflammatory burden index with 1-year major adverse cardiovascular events in patients with HFpEF after myocardial infarction: a single-center retrospective cohort study

**DOI:** 10.3389/fcvm.2026.1831654

**Published:** 2026-06-10

**Authors:** Xiaoyan Yin, Pan Chen, Boshi Liu, Lei Ren

**Affiliations:** 1 Fuyang People's Hospital, Bengbu Medical University Affiliated Fuyang Hospital, Fuyang, Anhui, China; 2 Fuyang People's Hospital, Anhui Medical University Affiliated Fuyang People's Hospital, Fuyang, Anhui, China

**Keywords:** C-reactive protein, heart failure with preserved ejection fraction, inflammatory burden index, major adverse cardiovascular events, myocardial infarction, neutrophil-to-lymphocyte ratio

## Abstract

**Background:**

Inflammatory activation may contribute to adverse remodeling and recurrent cardiovascular events after myocardial infarction (MI), but its prognostic value in patients with heart failure with preserved ejection fraction (HFpEF) remains uncertain. This study evaluated whether the Inflammatory Burden Index (IBI) was associated with 1-year major adverse cardiovascular events (MACE) in patients with HFpEF and documented MI.

**Method:**

In this single-centre retrospective cohort study, 625 patients hospitalized at Fuyang People's Hospital from January 2018 to December 2023 were screened. After 33 patients with incomplete follow-up or unavailable baseline data were excluded, 592 patients were included. IBI was calculated as C-reactive protein multiplied by the neutrophil-to-lymphocyte ratio. The Youden-derived IBI cutoff of 7.70 was used only for descriptive grouping and sensitivity analysis. The primary Cox model treated log-transformed IBI (logIBI) as a continuous predictor and adjusted for age, sex, hypertension, diabetes mellitus, LDL-C and soluble ST2. Discrimination, calibration and clinical usefulness were assessed using the C-index, 365-day AUC, Brier score, calibration slope, Hosmer-Lemeshow test and decision curve analysis.

**Results:**

During 1-year follow-up, 74 patients experienced MACE (12.5%). Higher logIBI was independently associated with 1-year MACE (HR 1.828, 95% CI 1.529–2.186, *P* < 0.001). In the sensitivity model, high IBI was also associated with increased MACE risk (HR 4.126, 95% CI 2.512–6.776, *P* < 0.001). Adding logIBI improved the 365-day AUC from 0.790 to 0.832 and increased the C-index from 0.776 to 0.817. The expanded model showed generally acceptable calibration, with a Brier score of 0.075, calibration slope of 0.938 and Hosmer-Lemeshow *P* value of 0.202. Decision curve analysis suggested higher net benefit after adding logIBI.

**Conclusion:**

In patients with HFpEF and documented MI, higher logIBI was independently associated with 1-year MACE and improved risk discrimination when added to a base clinical model. IBI may provide a practical admission-level measure of residual inflammatory burden, but external validation and prospective assessment are needed before clinical use.

## Introduction

Heart failure with preserved ejection fraction (HFpEF) is common among patients with coronary artery disease and is especially difficult to manage when it coexists with a history of myocardial infarction (MI). In this setting, ischemic injury, vascular inflammation, metabolic disease, renal vulnerability, and myocardial fibrosis often overlap. Contemporary pharmacologic evidence has improved the management landscape, including SGLT2 inhibition across the preserved or mildly reduced ejection-fraction spectrum and in patients at risk after acute MI, but residual post-MI vulnerability remains clinically meaningful (1–3). Evidence from anti-inflammatory and intensive lipid-lowering trials has also reinforced that inflammatory and lipid pathways remain important contributors to recurrent cardiovascular events despite standard care (4, 5).

Inflammation is not merely a background laboratory abnormality after MI. It participates in plaque instability, ischemia-reperfusion injury, microvascular dysfunction, and fibro-inflammatory remodeling. C-reactive protein (CRP) is the most widely used circulating inflammatory marker, and prior work has linked higher admission or peri-procedural CRP with adverse outcomes after acute MI and percutaneous coronary intervention (PCI) (6–8). The prognostic meaning of CRP can vary across metabolic phenotypes, and recent evidence suggests that body-mass or glycemic status may alter the relationship between CRP and cardiovascular outcomes (9). At the cellular level, the neutrophil-to-lymphocyte ratio (NLR) reflects the balance between innate immune activation and lymphocyte-mediated regulation and has been associated with short-term heart failure or adverse events after ST-segment elevation MI treated with primary PCI (10).

The Inflammatory Burden Index (IBI) integrates CRP with neutrophil and lymphocyte counts, thereby combining a protein-based acute-phase signal with a peripheral immune-cell profile. This integrated construction may be clinically useful in HFpEF after MI because inflammatory activation in this phenotype is often multifactorial rather than driven by a single biomarker. The present analysis therefore evaluated log-transformed IBI (logIBI) as the primary continuous inflammatory predictor, retained the Youden-derived IBI cutoff only for descriptive grouping and sensitivity analysis, and assessed discrimination, calibration, clinical utility, and model stability in a single-center post-MI HFpEF cohort.

## Materials and methods

### Study design and population

This was a single-center retrospective cohort study conducted at Fuyang People's Hospital. Consecutive adult patients hospitalized between January 2018 and December 2023 were screened if they had HFpEF and documented MI. HFpEF was defined as symptomatic heart failure with left ventricular ejection fraction (LVEF) ≥ 50%, consistent with the 2022 AHA/ACC/HFSA heart failure guideline (11). MI was identified according to contemporary clinical records and was interpreted in relation to the 2023 ESC acute coronary syndrome guideline framework (12). The index hospitalization was defined as the hospitalization from which baseline clinical variables and admission laboratory measurements were obtained. The cohort was analyzed as a documented post-MI HFpEF cohort rather than an acute MI-only cohort, because MI subtype, symptom-to-admission interval, and the exact interval between MI diagnosis and the index hospitalization were not consistently available in the final analytic dataset.

A total of 625 patients were initially screened. After exclusion of patients with incomplete follow-up or unavailable baseline data, 592 patients formed the final analysis cohort. Patients were followed for 1 year through outpatient records, electronic medical records, and telephone contact when available. The final analytic dataset did not contain the baseline records of the 33 patients lost to follow-up; therefore, formal comparison between lost patients and the final cohort could not be computed. This limitation was considered when interpreting selection bias.

Eligible patients were aged 18 years or older and had HFpEF with documented MI. Exclusion criteria included end-stage renal disease, hematologic disease, autoimmune disease, severe liver disease, severe infection, severe chronic obstructive pulmonary disease, malignancy, recent systemic glucocorticoid or immunosuppressive therapy, severe valvular disease, hypertrophic or restrictive cardiomyopathy, active myocarditis or pericarditis, uncorrected congenital heart disease, uncontrolled hyperthyroidism, life expectancy shorter than 1 year, inability to complete follow-up, or missing essential baseline data.

### Data collection and IBI calculation

Baseline variables were extracted from the hospital electronic medical record system. Demographic and clinical variables included age, sex, body mass index, smoking, drinking, hypertension, diabetes mellitus, coronary heart disease, hyperlipidemia, and NYHA functional class. Vital signs, routine laboratory variables, sST2, renal function indices, lipid parameters, thyroid function indices, inflammatory variables, and echocardiographic LVEF were collected at the index hospitalization. CRP was extracted in mg/L from routine hospital laboratory records. The de-identified analytic file retained CRP units and the admission measurement timepoint, but did not retain detailed assay-platform metadata or the local laboratory reference interval; therefore, these details were not imputed.

IBI was calculated from first available admission laboratory values as: IBI = CRP (mg/L) × neutrophil count (× 10^9^/L)/lymphocyte count (× 10^9^/L). Because the raw IBI distribution was right-skewed, logIBI was used as the primary continuous inflammatory predictor. The Youden-derived IBI cutoff of 7.70 was used only for descriptive grouping, Kaplan–Meier visualization, and sensitivity analysis. It was not treated as an externally validated clinical threshold.

### Endpoint definition

The primary outcome was 1-year MACE, defined as the first occurrence of cardiac death, non-fatal recurrent MI, or readmission for heart failure during follow-up. Recurrent MI was identified from documented clinical diagnosis compatible with guideline-based MI assessment, including recurrent ischemic symptoms or objective evidence of new myocardial injury when available. Heart-failure readmission was defined as unplanned hospitalization for worsening heart-failure symptoms requiring treatment escalation.

### Statistical analysis

Statistical analyses were performed using R. Continuous variables were summarized as mean ± standard deviation or median (interquartile range), and categorical variables as number and percentage. Between-group comparisons used the *t*-test, Mann–Whitney *U* test, chi-square test, or Fisher exact test as appropriate. All tests were two-sided, with *P* < 0.05 considered statistically significant.

To avoid unstable selection caused by mathematically overlapping inflammatory variables, the modeling strategy did not simultaneously retain CRP, NLR, IBI, logIBI, and the high-IBI indicator as interchangeable inflammatory constructs in the final multivariable model. LASSO Cox regression with 10-fold cross-validation was used as a screening step for non-overlapping clinical covariates. The primary multivariable Cox model included age, sex, hypertension, diabetes mellitus, LDL-C, sST2, and logIBI. A binary high-IBI indicator based on the Youden-derived cutoff was evaluated only in a sensitivity model. The proportional hazards assumption was assessed using Schoenfeld residuals, and multicollinearity was evaluated using variance inflation factors (VIFs). Restricted cubic splines were used to explore dose-response and nonlinearity for logIBI.

Model performance was evaluated using the C-index, 365-day ROC analysis, Brier score, calibration intercept and slope, grouped calibration plots, Hosmer–Lemeshow testing, and decision curve analysis. Incremental value after adding logIBI was described using apparent net reclassification improvement and integrated discrimination improvement. The base clinical model included age, sex, hypertension, diabetes mellitus, LDL-C, and sST2; the expanded model added logIBI. Missingness was summarized for all variables included in the analytic dataset; after final extraction, variables used in the analysis had no missing values.

## Results

### Baseline characteristics

Among 592 patients in the final cohort, 74 experienced 1-year MACE (12.5%). The Youden-derived IBI cutoff was 7.70, yielding 179 patients in the high-IBI group and 413 in the low-IBI group. Patients in the high-IBI group were older and had higher frequencies of hypertension and diabetes mellitus. They also had higher neutrophil counts, CRP, NLR, IBI, and logIBI, while lymphocyte counts and eGFR were lower. LVEF and sST2 did not differ significantly by IBI group. Baseline characteristics are summarized in Table 1.

**Table 1:** Baseline characteristics according to IBI group.

Domain	Variable	High IBI (*n* = 179)	Low IBI (*n* = 413)	*P* value
General information	Age, years	62.00 (52.50, 72.00)	58.00 (50.00, 70.00)	0.032
General information	Male sex	92 (51.4%)	200 (48.4%)	0.566
General information	BMI, kg/m²	25.50 (23.25, 27.55)	24.70 (22.40, 27.50)	0.081
Medical history and disease severity	Hypertension: no	118 (65.9%)	339 (82.1%)	< 0.001
Medical history and disease severity	Hypertension: yes	61 (34.1%)	74 (17.9%)	< 0.001
Medical history and disease severity	Diabetes mellitus: no	126 (70.4%)	353 (85.5%)	< 0.001
Medical history and disease severity	Diabetes mellitus: yes	53 (29.6%)	60 (14.5%)	< 0.001
Medical history and disease severity	Smoking: no	142 (79.3%)	348 (84.3%)	0.180
Medical history and disease severity	Smoking: yes	37 (20.7%)	65 (15.7%)	0.180
Medical history and disease severity	Drinking: no	145 (81.0%)	337 (81.6%)	0.956
Medical history and disease severity	Drinking: yes	34 (19.0%)	76 (18.4%)	0.956
Vital signs and imaging	LVEF, %	56.00 (52.00, 59.00)	56.00 (53.00, 59.00)	0.302
Vital signs and imaging	SBP, mmHg	131.00 (119.00, 143.00)	132.00 (120.00, 143.00)	0.997
Vital signs and imaging	DBP, mmHg	80.04 ± 12.10	78.26 ± 12.13	0.102
Laboratory variables	WBC, × 10^9^/L	7.26 ± 1.70	7.19 ± 1.80	0.685
Laboratory variables	Neutrophils, × 10^9^/L	5.94 ± 1.25	4.54 ± 1.33	< 0.001
Laboratory variables	Lymphocytes, ×10⁹/L	1.27 (0.89, 1.60)	2.09 (1.67, 2.48)	< 0.001
Laboratory variables	Platelets, × 10^9^/L	209.34 ± 47.71	210.95 ± 51.25	0.712
Laboratory variables	sST2, ng/mL	37.00 (27.00, 48.00)	36.00 (28.00, 46.00)	0.709
Laboratory variables	ALT, U/L	25.00 (17.00, 36.00)	27.00 (18.00, 40.00)	0.217
Laboratory variables	AST, U/L	26.00 (19.00, 35.00)	24.00 (16.00, 36.00)	0.255
Laboratory variables	Creatinine, μmol/L	96.00 (74.00, 117.50)	87.00 (68.00, 109.00)	0.011
Laboratory variables	eGFR, mL/min/1.73 m²	84.60 (72.15, 97.75)	89.30 (78.00, 102.40)	0.002
Laboratory variables	Uric acid, μmol/L	365.12 ± 98.06	359.65 ± 95.08	0.530
Laboratory variables	Total cholesterol, mmol/L	4.70 ± 0.92	4.73 ± 0.86	0.658
Laboratory variables	Triglyceride, mmol/L	1.37 (0.95, 1.81)	1.29 (0.93, 1.81)	0.737
Laboratory variables	LDL-C, mmol/L	2.79 ± 0.71	2.80 ± 0.77	0.824
Laboratory variables	HDL-C, mmol/L	1.11 ± 0.21	1.11 ± 0.22	0.995
Laboratory variables	Sodium, mmol/L	140.00 (138.00, 143.00)	140.00 (138.00, 143.00)	0.621
Laboratory variables	Potassium, mmol/L	4.20 (3.90, 4.40)	4.10 (3.80, 4.30)	0.046
Inflammatory indices	CRP, mg/L	4.21 (2.92, 6.40)	1.01 (0.53, 1.79)	< 0.001
Inflammatory indices	NLR	4.48 (3.44, 6.88)	2.17 (1.56, 3.10)	< 0.001
Inflammatory indices	IBI	18.73 (12.22, 33.02)	2.41 (1.07, 4.55)	<0.001
Inflammatory indices	logIBI	3.08 ± 0.79	0.74 ± 0.96	< 0.001

Continuous variables are shown as mean ± SD or median (interquartile range); categorical variables are shown as *n* (%). CRP, C-reactive protein; IBI, inflammatory burden index; NLR, neutrophil-to-lymphocyte ratio; sST2, soluble ST2; eGFR, estimated glomerular filtration rate.

### Variable selection and Cox models

The variable-selection strategy avoided simultaneous final-model inclusion of overlapping inflammatory constructs. LASSO screening supported retention of clinically relevant variables, but the final primary Cox model used logIBI as the single inflammatory burden representation. This strategy addressed the mathematical overlap among CRP, NLR, IBI, logIBI, and the high-IBI indicator, while allowing CRP, NLR, and IBI to be compared descriptively in discrimination analyses.

**Figure 1:**
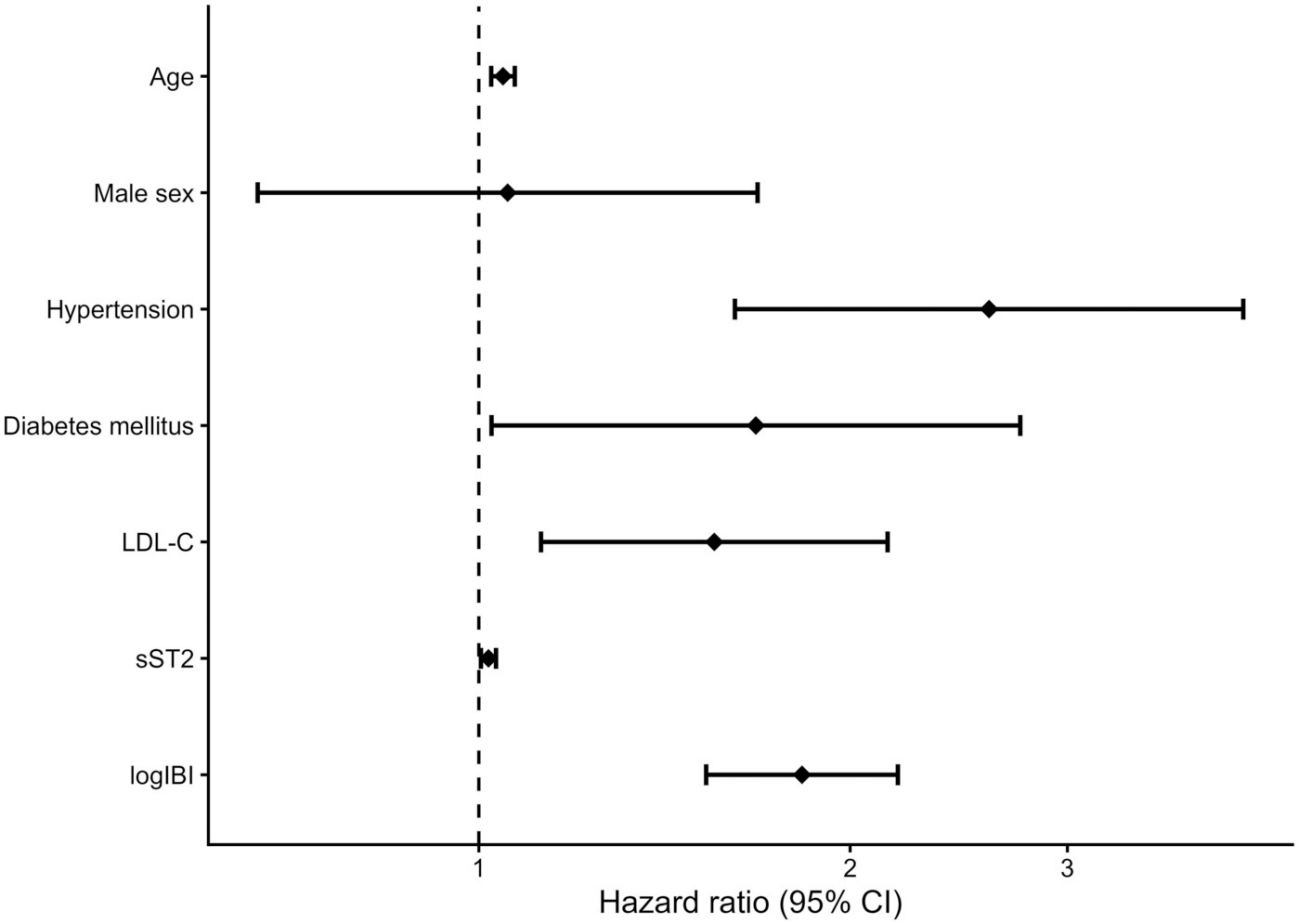
Multivariable Cox regression for 1-year MACE. Hazard ratios are shown with 95% confidence intervals. The primary model included age, sex, hypertension, diabetes mellitus, LDL-C, sST2, and logIBI.

In the primary Cox model, logIBI was independently associated with 1-year MACE after adjustment for age, sex, hypertension, diabetes mellitus, LDL-C, and sST2 (HR 1.828, 95% CI 1.529–2.186, *P* < 0.001). Age, hypertension, diabetes mellitus, LDL-C, and sST2 were also associated with outcome, whereas sex was not. In the sensitivity model using the high-IBI indicator, high IBI remained associated with increased MACE risk (HR 4.126, 95% CI 2.512–6.776, *P* < 0.001). The Cox regression results are summarized in Table 2. The VIF range in the primary model was 1.01–1.09, supporting absence of clinically relevant multicollinearity. Schoenfeld residual testing did not suggest violation of the proportional hazards assumption. The multivariable Cox regression results are shown in Figure 1.

**Table 2:** Multivariable cox regression for 1-year MACE.

Variable	*β*	SE	HR	95% CI	*P* value
Age	0.045	0.011	1.046	1.023–1.069	< 0.001
Male sex	0.054	0.238	1.055	0.662–1.683	0.821
Hypertension	0.952	0.242	2.592	1.613–4.166	< 0.001
Diabetes mellitus	0.517	0.252	1.677	1.024–2.747	0.040
LDL-C	0.440	0.165	1.552	1.123–2.145	0.008
sST2	0.018	0.007	1.018	1.004–1.033	0.012
logIBI	0.603	0.091	1.828	1.529–2.186	< 0.001

Sensitivity analysis using the Youden-derived high IBI group showed HR 4.126 (95% CI 2.512–6.776), *P* < 0.001. The primary model included age, sex, hypertension, diabetes mellitus, LDL-C, sST2, and logIBI.

### Dose-response pattern of logIBI

**Figure 2:**
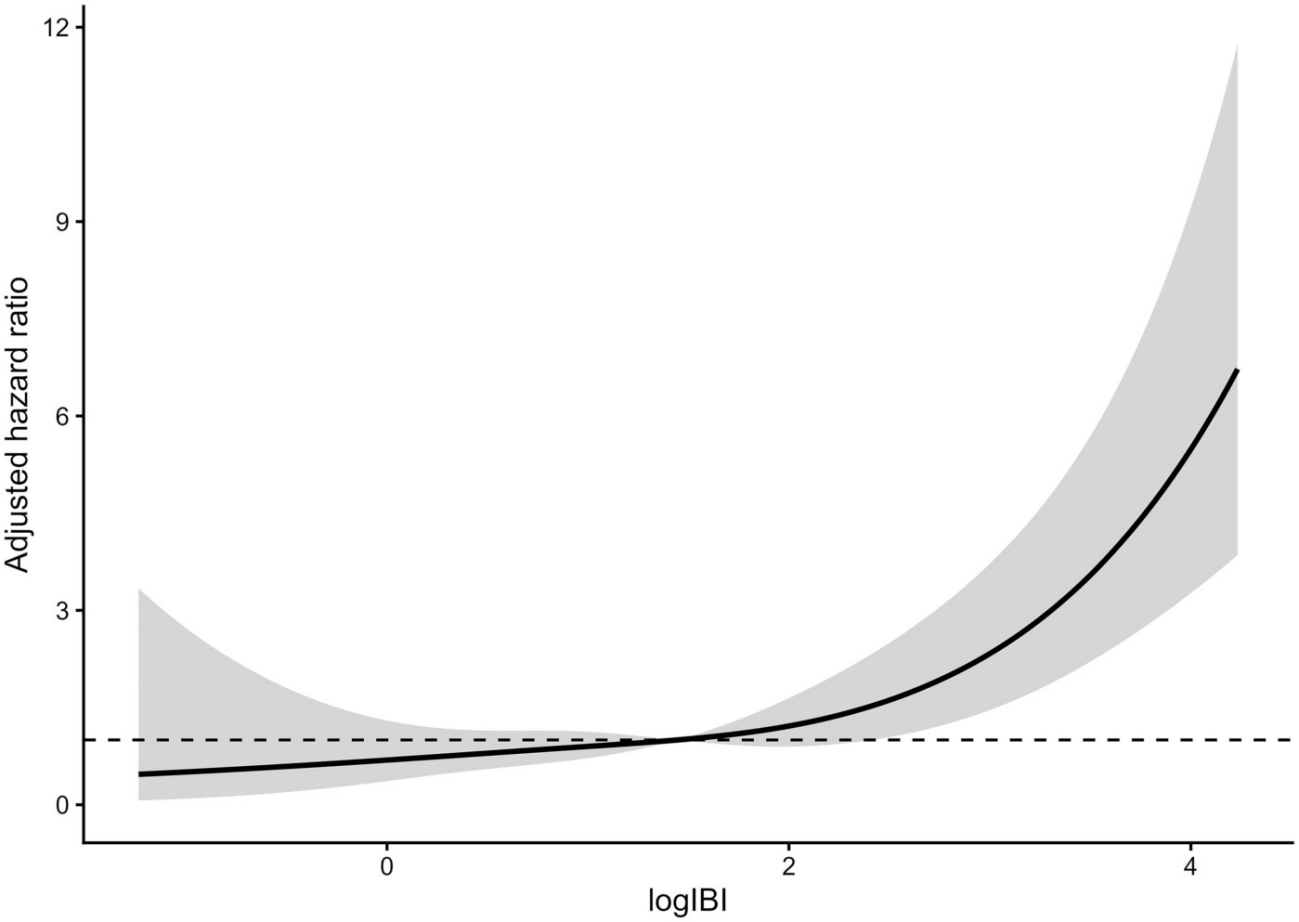
Restricted cubic spline analysis for the association between logIBI and 1-year MACE. The model was adjusted for the primary clinical covariates. The solid line indicates the adjusted hazard ratio and the shaded area indicates the 95% confidence interval.

Restricted cubic spline analysis showed a significant overall association between logIBI and MACE (*P* < 0.001), without strong evidence of nonlinearity (*P* for nonlinearity = 0.139). The risk increased gradually with higher logIBI, while uncertainty widened in the upper tail of the distribution. The dose-response pattern is shown in Figure 2.

### IBI grouping and event-free survival

**Figure 3:**
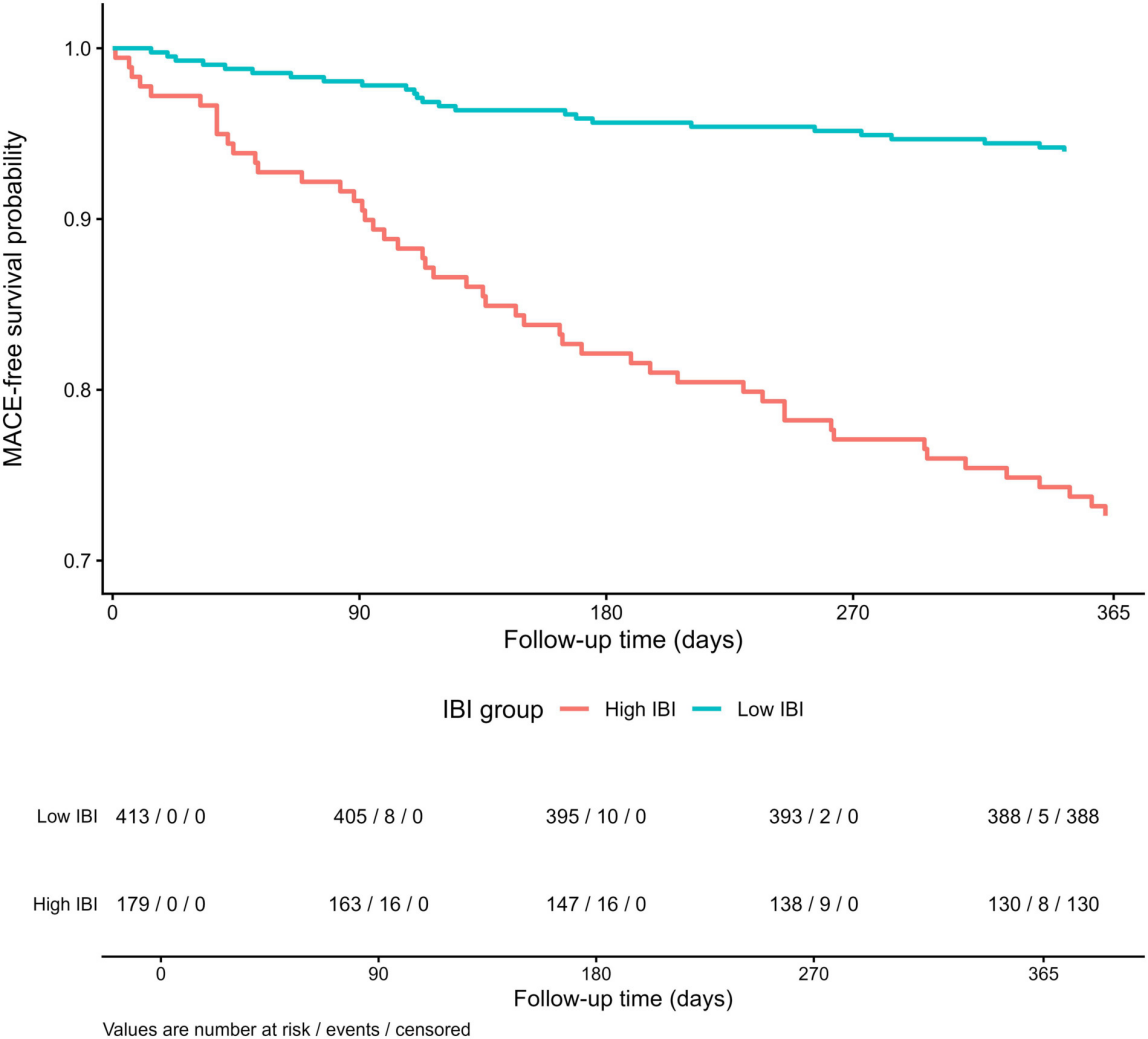
Kaplan–meier curves for 1-year MACE-free survival according to IBI group. Values below the plot are number at risk/interval events/administrative or interval censoring at prespecified time points.

Kaplan–Meier analysis showed clear separation of 1-year MACE-free survival curves between IBI groups. At 365 days, the MACE-free survival probability was 0.726 in the high-IBI group and 0.939 in the low-IBI group. The accompanying risk table displays number at risk, interval events, and administrative or interval censoring. The Kaplan–Meier curves are shown in Figure 3.

### Model performance, calibration, and clinical utility

**Figure 4:**
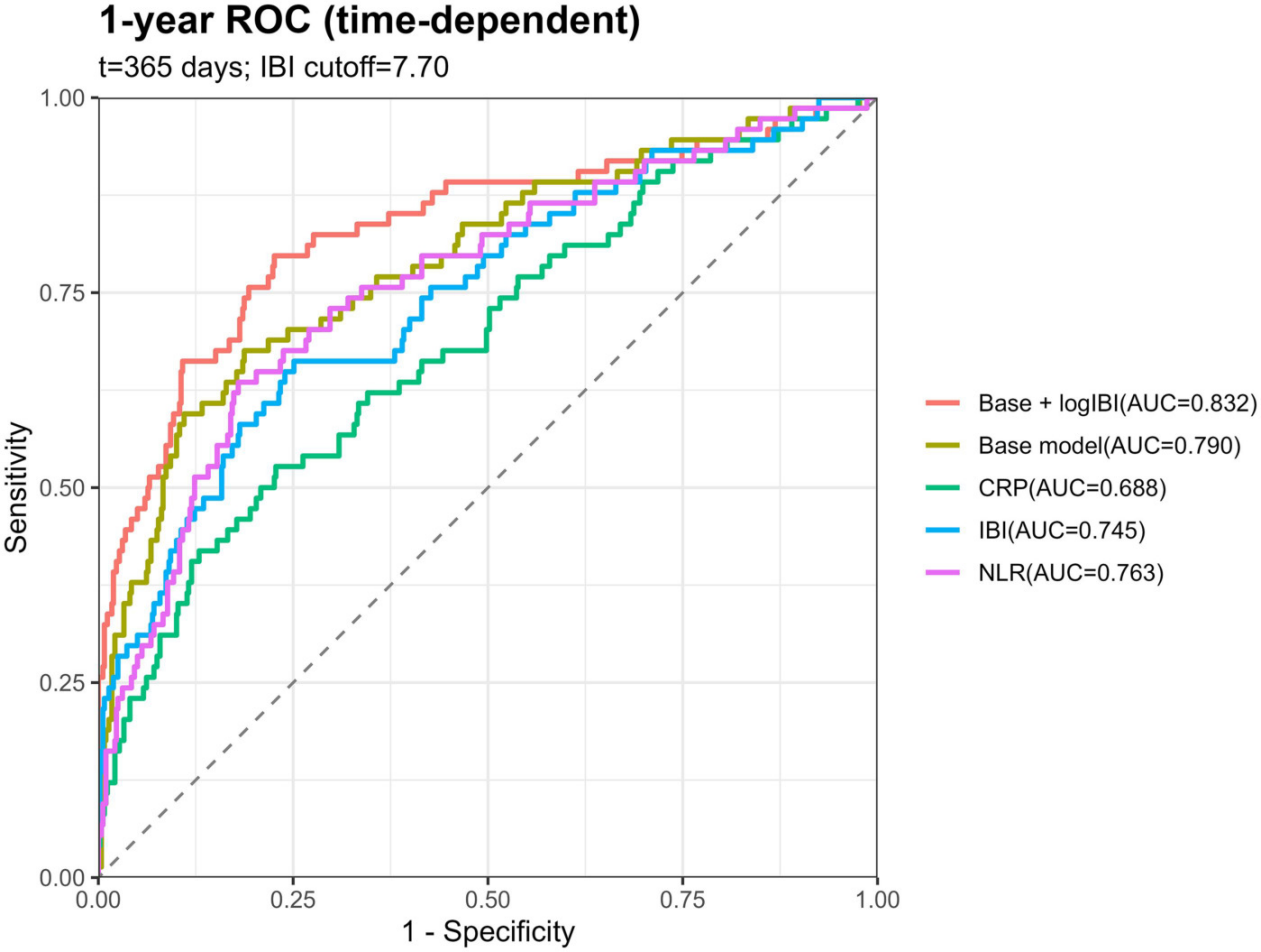
Time-dependent ROC curves for 1-year MACE at 365 days. The Youden-derived IBI cutoff for descriptive grouping was 7.70.

At 365 days, the AUCs were 0.745 for IBI, 0.688 for CRP, 0.763 for NLR, 0.790 for the base clinical model, and 0.832 for the base clinical model plus logIBI. These results indicate that IBI alone did not outperform NLR as a single marker, but adding logIBI improved discrimination beyond the base clinical model. The C-index increased from 0.776 for the base clinical model to 0.817 after adding logIBI. The time-dependent ROC curves are shown in Figure 4.

**Figure 5:**
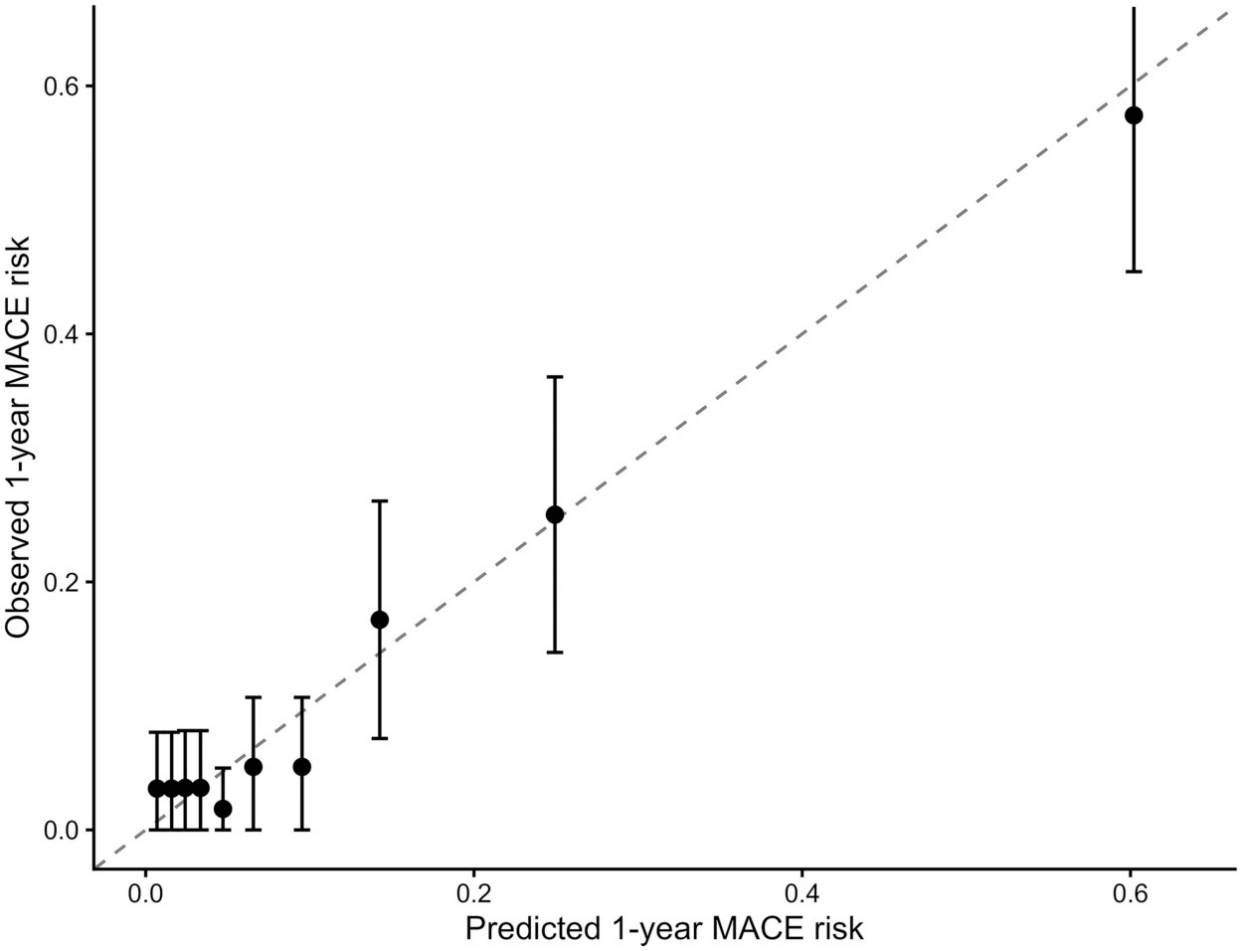
Calibration plot for the base clinical model plus logIBI using 365-day absolute predicted risk. Points represent deciles of predicted risk; vertical bars indicate approximate 95% confidence intervals. The dashed line indicates ideal calibration.

**Figure 6:**
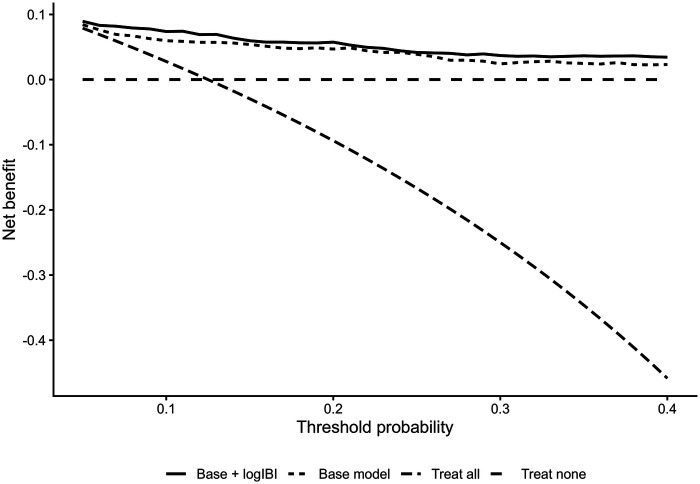
Decision curve analysis for 1-year MACE. Net benefit is shown across clinically relevant threshold probabilities for the base clinical model, the base clinical model plus logIBI, treat-all, and treat-none strategies.

Calibration of the base clinical model plus logIBI was generally acceptable using 365-day absolute predicted risk. The Brier score was 0.075, the calibration intercept was −0.037, the calibration slope was 0.938, and the Hosmer–Lemeshow *P* value was 0.202. Grouped calibration was close to the ideal line overall, although confidence intervals were wider in higher-risk strata. The grouped calibration plot is shown in Figure 5. Decision curve analysis suggested that the model including logIBI had higher net benefit than the base clinical model across clinically relevant threshold probabilities. Model performance and incremental predictive metrics are summarized in Table 3. Apparent reclassification metrics after adding logIBI were NRI_cat 0.127, NRI_cont 0.629, and IDI 0.109. Decision curve analysis is shown in Figure 6.

**Table 3:** Discrimination, calibration, and incremental value of the base clinical model plus logIBI.

Domain	Metric	Estimate
AUC at 365 days	IBI	0.745
AUC at 365 days	CRP	0.688
AUC at 365 days	NLR	0.763
AUC at 365 days	Base clinical model	0.790
AUC at 365 days	Base clinical model + logIBI	0.832
C-index	Base clinical model	0.776
C-index	Base clinical model + logIBI	0.817
Calibration	Brier score	0.075
Calibration	Calibration intercept	−0.037
Calibration	Calibration slope	0.938
Calibration	Hosmer–Lemeshow *P* value	0.202
Incremental value	NRI_cat	0.127
Incremental value	NRI_cont	0.629
Incremental value	IDI	0.109

NRI and IDI are apparent estimates from the final dataset and should be interpreted as exploratory internal performance measures.

## Discussion

This study found that higher inflammatory burden, represented primarily by logIBI, was independently associated with 1-year MACE in patients with HFpEF and documented MI. The association persisted after adjustment for age, sex, hypertension, diabetes mellitus, LDL-C, and sST2. Importantly, the main inference did not depend on a single data-derived dichotomous cutoff. The Youden-derived IBI threshold was retained for descriptive stratification and sensitivity analysis, while the primary analysis was based on a continuous logIBI model. This approach is more conservative and clinically interpretable, particularly in a retrospective cohort where a threshold derived and tested in the same population is vulnerable to optimism.

The findings fit with the broader biology of post-MI HFpEF. After MI, inflammatory activation participates in infarct healing but may become maladaptive when excessive or prolonged. Neutrophil activation can amplify endothelial injury, microvascular obstruction, and oxidative stress, whereas lymphocyte depletion may reflect impaired immune regulation. CRP, in turn, reflects hepatic acute-phase response driven largely by interleukin-6 signaling. In a patient with HFpEF, this systemic inflammatory environment may aggravate vascular stiffness, coronary microvascular dysfunction, extracellular matrix turnover, and myocardial fibrosis. The final pathway is not simply recurrent ischemia; it may involve a gradual shift toward diastolic dysfunction, impaired reserve, and susceptibility to decompensated heart failure. IBI is biologically plausible because it joins these protein and cellular inflammatory signals rather than treating them as isolated measurements.

The comparative marker results deserve careful interpretation. IBI alone had a 365-day AUC of 0.745, which was higher than CRP but lower than NLR. Therefore, the present data do not support a claim that IBI universally outperforms NLR as a single discriminator. The clinically relevant finding is different: when logIBI was added to a base model containing established clinical and biomarker covariates, the AUC increased from 0.790 to 0.832 and the C-index increased from 0.776 to 0.817. Previous studies have reported that NLR and NLR-based combinations may predict adverse outcomes after STEMI or PCI, and a meta-analysis has supported the prognostic relevance of NLR while emphasizing heterogeneity across populations and endpoints (13, 14). The present results should be read in that context. IBI may be most useful not as a replacement for other inflammatory indices, but as an admission-level marker that complements conventional clinical risk assessment.

The role of sST2 in the model also has clinical rationale. sST2 has been linked to myocardial stress, fibrosis, inflammation, and adverse outcomes across heart-failure phenotypes (15, 16). In STEMI patients undergoing PCI, higher sST2 has been associated with slow-flow/no-reflow and short-term adverse events, suggesting that it may capture ischemia-reperfusion injury and myocardial stress beyond systemic inflammation alone (17, 18). Recent synthesis in chronic heart failure likewise supports the prognostic value of circulating sST2, although the strength of association may depend on assay timing, phenotype, and outcome definition (19). Adjusting for sST2 therefore helped test whether logIBI contributed information beyond an established fibrosis- and stress-related biomarker.

Renal vulnerability is another relevant component of risk in this cohort. eGFR is associated with prognosis in HFpEF, and renal impairment can affect treatment selection, inflammatory state, and post-MI recovery (20). Trial analyses in HFpEF/HFmrEF have shown that eGFR trajectories after SGLT2 inhibition should be interpreted in a therapeutic context rather than viewed as simple renal injury signals (21). Large contemporary data in MI patients further show that chronic kidney disease remains associated with differences in management and worse outcomes, even in the modern era of invasive and medical therapy (22, 23). Although eGFR did not enter the final primary model, differences in renal function by IBI group support the need to interpret inflammatory biomarkers together with cardiorenal risk.

External evidence for IBI is growing but remains heterogeneous. Nationally representative analyses have related IBI to long-term mortality, while coronary heart disease data have associated higher IBI with adverse cardiovascular or cerebrovascular outcomes (24, 25). IBI has also been studied in atrial fibrillation recurrence after catheter ablation, readmission after PCI, and heart failure risk in NHANES data, suggesting that it may reflect a broader systemic vulnerability rather than a disease-specific signal (26–28). Studies in acute ischemic stroke after endovascular thrombectomy extend this idea to non-cardiac vascular injury (29). Related indices, including CRP-albumin-lymphocyte index, lymphocyte-to-CRP ratio, and systemic immune-inflammation index, have also shown associations with acute coronary syndrome severity, contrast-induced kidney injury, or AMI outcomes (30–32). The common theme is that combined inflammatory and immune-cell measures can carry prognostic information, but each index requires validation in the specific clinical context where it is intended to be used.

The clinical implication of the present work is modest but practical. IBI can be calculated from routine admission CRP, neutrophil count, and lymphocyte count without specialized testing. In settings where HFpEF patients with prior MI are followed after discharge, a high inflammatory burden may identify patients who warrant closer surveillance, more careful assessment of residual congestion, renal function, lipid control, and adherence to contemporary cardioprotective therapy. Whether IBI can be used to select patients for anti-inflammatory therapies such as colchicine remains speculative, but the present findings support the hypothesis that residual inflammatory burden identifies higher-risk individuals. It would be premature to use IBI alone to guide treatment escalation. A more reasonable near-term role is as a low-cost stratification marker that may complement established clinical assessment.

Several limitations should be considered. First, the study was single-center and retrospective, and causality cannot be inferred. Second, the final analytic dataset did not contain the baseline records of patients lost to follow-up, so formal attrition comparison could not be performed. Third, key treatment-related and disease-severity variables, including STEMI/NSTEMI classification, symptom-to-admission time, reperfusion strategy, PCI timing and success, discharge medications, atrial fibrillation, and previous heart failure, were not consistently available in the final dataset. Residual confounding by disease severity and post-MI management is therefore possible. Fourth, CRP assay-platform details and local reference intervals were not retained in the de-identified analytic file, limiting laboratory reproducibility. Fifth, IBI was measured only at baseline; dynamic inflammatory trajectories may provide stronger prognostic information. Finally, the IBI cutoff was derived from the same cohort and should not be treated as a validated threshold. External validation in multicenter cohorts and prospective testing of IBI-guided follow-up strategies are needed.

## Conclusion

In patients with HFpEF and documented MI, higher logIBI was independently associated with 1-year MACE and improved discrimination when added to a base clinical model. The Youden-derived IBI cutoff was useful for descriptive risk stratification but requires external validation before clinical adoption. IBI may provide a practical admission-level measure of residual inflammatory burden, but its role in guiding treatment intensity should be tested prospectively.

## Data Availability

The original contributions presented in the study are included in the article/Supplementary Material, further inquiries can be directed to the corresponding author.
