# Supplementary material for: Association of the inflammatory burden index with 1-year major adverse cardiovascular events in patients with HFpEF after myocardial infarction: a single-center retrospective cohort study
Source: Front Cardiovasc Med. 2026 Jun 10;13:1831654. doi: 10.3389/fcvm.2026.1831654 (PMC13290569; doi:10.3389/fcvm.2026.1831654)
Supplement: Supplementary file 1 [file Datasheet1.pdf]

Supplementary material

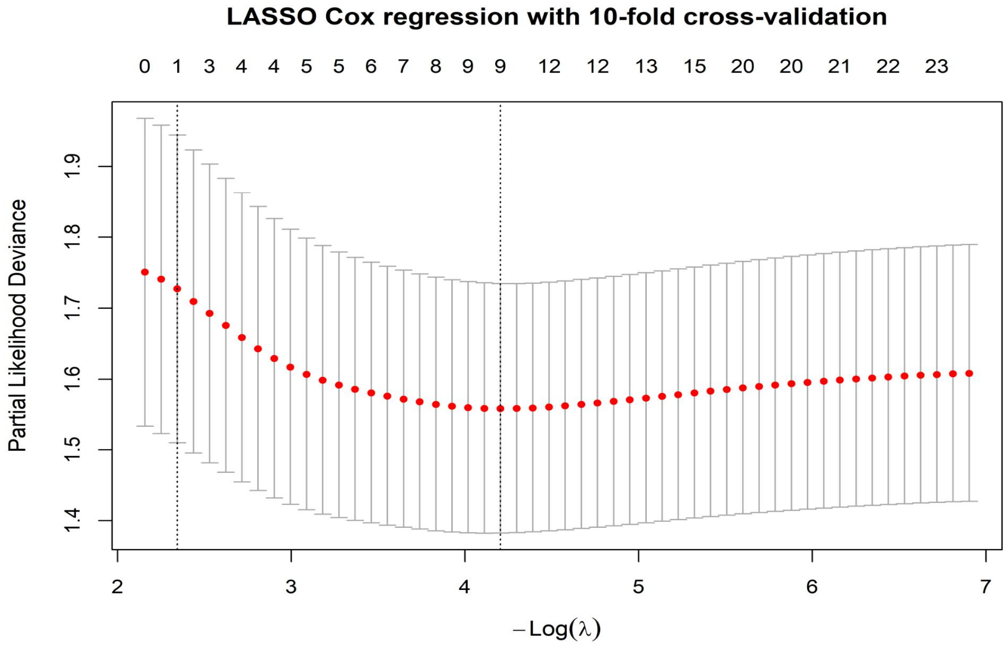

Supplementary Figure S1. LASSO Cox regression with 10-fold cross-validation.

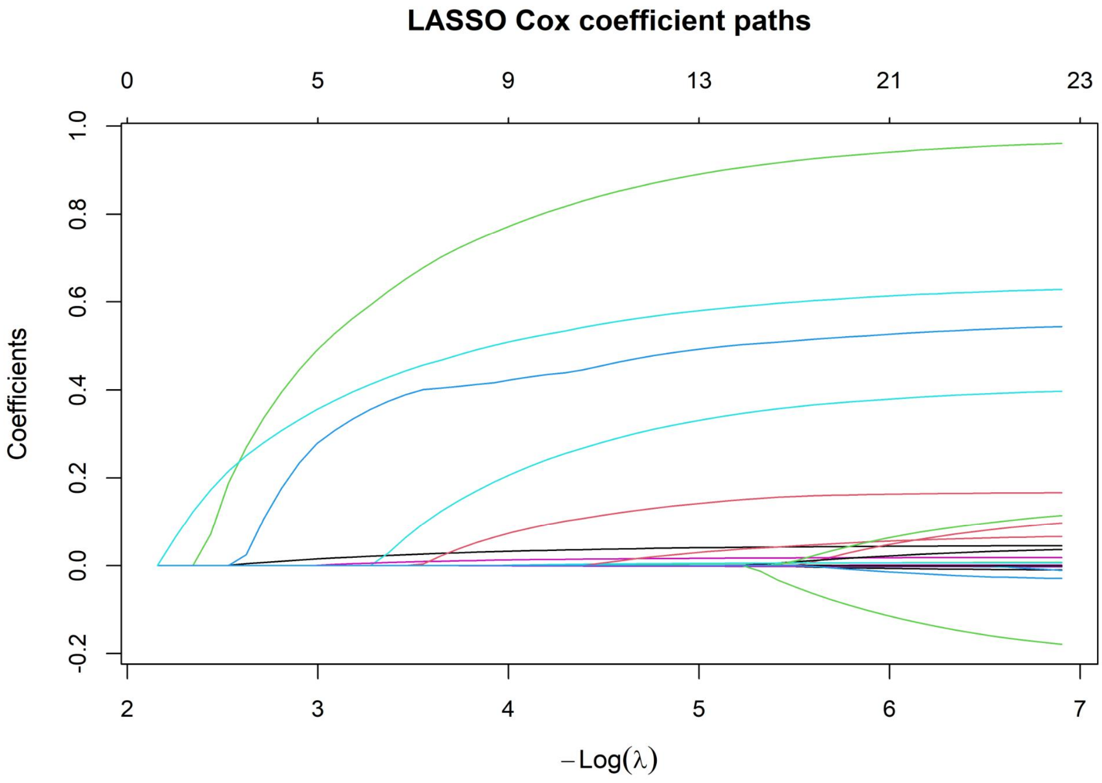

Supplementary Figure S2. LASSO Cox coefficient paths.
